# Quantifying the impact of disease severity changes on the burden of blindness: A global decomposition analysis

**DOI:** 10.7189/jogh.14.04248

**Published:** 2024-11-01

**Authors:** Jianqi Chen, Xiaohong Chen, Yingting Zhu, Zhidong Li, Xuhao Chen, Xu Cao, Yangyang Li, Yuwen Wen, Liyan Liu, Yue Xiao, Jinan Zhan, Haishun Huang, Yingfeng Zheng, Yiqing Li, Yantao Wei, Yehong Zhuo

**Affiliations:** State Key Laboratory of Ophthalmology, Zhongshan Ophthalmic Center, Sun Yat-sen University, Guangdong Provincial Key Laboratory of Ophthalmology and Visual Science, Guangzhou, China

## Abstract

**Background:**

Despite the significant impact of blindness on the affected individuals’ quality of life, its burden has not been assessed according to temporal cause-specific changes in severity, impeding our ability to evaluate the impact of blindness on population health accurately. Therefore, we aimed to comprehensively quantify the changes in cause-specific blindness burden according to changes in disease severity for 18 causes of blindness.

**Methods:**

For this cross-sectional population-based study, we derived data on prevalence, disability-adjusted life-years (DALYs), and population size between 1990 and 2019 from the Global Burden of Disease 2019 study. Using the decomposition method, we attributed changes in total DALYs to population growth, population ageing, and changes in prevalence rate and disease severity between 1990 and each subsequent year globally, regionally, nationally, and by sex, cause, and sociodemographic index (SDI). The absolute and relative contributions to the variation in blindness-related DALYs between 1990 and each year from 1991 to 2019 then served as a measure of changes in disease severity.

**Results:**

Changes in disease severity from 1990 to 2019 were associated with 15 165.11 DALYs in men and 20 639.32 DALYs in women. We observed disease severity increases in most countries/territories, with attributable DALY proportions ranging from −0.07% to 1.30% in men and from −0.06% to 1.73% in women. Notably, both attributable proportions and DALYs were greater in women than men. The largest increases in attributable DALYs were observed for cataracts, refraction disorders, and glaucoma globally; age-related macular degeneration in high-SDI countries; and trachoma and retinopathy of prematurity in lower-SDI countries.

**Conclusions:**

Growth in the burden of cause-specific blindness due to increased disease severity reflects the lag of healthy vision life behind increasing life expectancy, necessitating the implementation of preventive and long-term therapeutic measures focussed on improving visual outcomes.

Blindness is estimated to affect more than 36 million individuals globally, resulting in significant challenges for them and their families, as well as their communities and society in general. Numerous studies on the prevalence and patterns of blindness have yielded significant insights for policymakers and researchers, enabling coordinated efforts that have resulted in a discernible reduction in the age-standardised prevalence of blindness globally [1]. Bourne et al. [2] underscore the critical global challenge of blindness, highlighting that, despite a reduction in the age-standardised prevalence over the past three decades, the absolute number of individuals affected has significantly increased due to population growth and ageing. This finding stresses the pressing need for enhanced global vision health care strategies to adapt to demographic shifts. Li et al. [3], meanwhile, evaluated the global burden of blindness and vision loss, quantified in years lived with disability (YLDs), and observed a significant health loss due to vision impairment and underscores the necessity of efficient health-service planning and resource distribution [3]. Yang et al. [4] expanded on their findings by noting the rise in global disability-adjusted life years (DALYs), despite the marginally reduced incidence of blindness and vision loss, indicating persistent difficulties in effectively addressing the impact of eye diseases. These difficulties emphasise the critical need for more accurate interventions specifically designed according to disease severity. Targeted interventions are particularly crucial for addressing the severity among distinct causes of blindness to lessen its broader social effects.

However, most prior studies have not decomposed the effect of the severity of blindness-causing diseases on disease burden, precluding accurate evaluation of the impact of blindness on population health. To bridge this gap, we utilised a four-factor decomposition analysis, focussing on the nuanced role of disease severity. This choice stems from the method's ability to isolate the impact of disease severity, a critical yet often overlooked determinant of blindness burden. By employing this approach, we aimed to understand how severity changes dynamically influence DALYs, thus guiding more precise public health strategies [5].

Conceptually, DALYs emphasise the magnitude and consequences of illness within the population by comprehensively describing the societal impact of disease burden, including early mortality and long-term repercussions of illness and injury [6]. The primary focus of this metric is social justice, including the correlation between health conditions caused by diseases and the subsequent impact on individuals’ welfare, subjective well-being, and overall quality of life. In practice, it acts as a composite measure that integrates years of life lost and years lived with disability. The values assigned to these components can vary depending on specific estimates of prevalence, incidence, and life expectancy [7,8]. This variability introduces complexity when attempting to draw and compare conclusions.

We employed a four-factor decomposition analysis to examine variations in DALYs attributed to population growth, ageing, shifts in disease prevalence rates, and alterations in disease severity [9]. According to Das Gupta's [5] methodology, this approach enables the quantification of the impact of these elements on aggregate disease burden. Our objective was to elucidate the proportional influences of disease severity changes on blindness-related DALYs, providing insights for informing public health strategies and interventions. Adopting this decomposition technique allows a distinctive perspective on the relationships between factors influencing global health outcomes. Our findings could improve our understanding of the impact of the severity of blindness-causing diseases on disease burden and facilitate the enhancement of global vision health care systems by aiding decision-making regarding strategic priorities and resource allocation.

## METHODS

### Study population and data collection

We obtained repeated, cross-sectional, population-related, and blindness-related data from the Global Burden of Disease 2019 study (GBD 2019) [10], which evaluated the global burden of 369 diseases and injuries from 1990 to 2019, including the burden of blindness [11]. Data on the burden of blindness were derived from population-based research assessing visual acuity, including peer-reviewed publications and grey literature and surveys containing unit-record data. The source GBD study adjusted the data using several methods to ensure uniformity [11]. First, in cases where studies only provided data for both sexes, meta-regression with Bayesian priors, regularisation, and trimming was employed to disaggregate these data points into sex-specific data points. In cases where studies provided data on visual acuity across several thresholds (e.g. <6/60 instead of distinct thresholds for severe vision loss and blindness), logit difference adjustment meta-regression was conducted using data from studies that reported vision loss based on both severity levels. In cases where the best-corrected vision loss was documented but not the presenting vision loss, data points were crosswalked via logit difference meta-regression, which provided projected data points on presenting vision loss for research that did not expressly record such information. In cases where the data points covered an age range of more than 25 years, an algorithm was used to divide the data into five-year age groups using the age pattern of the super region, as determined by the DisMod-MR model. Following these adjustments, the GBD 2019 used the DisMod-MR, version 2.1 model (Health Metrics and Evaluation, University of Washington, Seattle, WA, USA) to estimate the overall and cause-specific burden of blindness.

We based our definitions of blindness and its causes on those used in the GBD 2019 [11]. Specifically, we defined blindness as a visual acuity of less than 3/60 according to a Snellen Chart or less than 10% visual field around the central fixation point. We thereby considered a total of 18 causes of blindness in our analysis (Table S1 in the **Online Supplementary Document**).

The burden of blindness was estimated based on its prevalence and related DALYs [12]. We assessed the burden of blindness by sex, age, cause, and sociodemographic index (SDI) at the global, regional, and national levels across the 204 countries and territories covered by the GBD 2019. The SDI is a comprehensive measure of the social and economic factors impacting health outcomes in specific locations. Its value ranges from 0 to 1 and is determined by calculating the geometric mean of the total fertility rate, average years of education, and lag-distributed income per capita. A value of 0 corresponds to the lowest number of years of education, lowest per capita income, and highest fertility rate. In this analysis, we adopted the GBD 2019 regional classification, which divided the 204 countries and territories into five sociodemographic groups based on SDI quintiles and 21 geographic regions based on epidemiological commonality and geographic hierarchy [11].

Since the GBD database contains anonymised and aggregated publicly available data on its website, the Medical Ethics Committee of the Zhongshan Ophthalmic Center at Sun Yat-sen University, Guangzhou, China, approved this study and waived the requirement for obtaining informed consent (approval number: 2023KYPJ095). We reported our findings per the Guidelines for Accurate and Transparent Health Estimates Reporting statement.

### Statistical analysis

We retrieved the number of prevalent cases, DALYs, and their respective rates directly from the GBD 2019 database [10]. We then presented the rates per 100 000 population. Based on the GBD method, the 95% uncertainty interval (UI) reflects the 25th and 975th values out of 1000 draws and is calculated for all estimates. We employed the decomposition method reported by Chang et al. [9] to attribute differences in blindness-related DALYs to four factors: population age structure, population size, age-standardised prevalence rates, and disease severity. We selected this method for its unique ability to dissect and quantify the individual effects of these key determinants on disease burden. Unlike conventional analytic approaches, the decomposition method offers a comprehensive framework for understanding the multifaceted drivers of health outcomes in a global context, thereby facilitating the identification of targeted areas for public health intervention by isolating the contribution of disease severity from overall health trends. It is especially appropriate for our study’s focus on global disease burden, where understanding the separate impact of disease severity is crucial for devising effective health policies and programmes.

We used the variation in total blindness-related DALYs and subgroup DALYs between 1990 and each year from 1991 to 2019 to determine the absolute and relative contributions of the four factors. as follows:



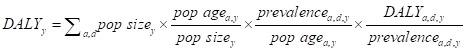


In this formula, the variable *a* represents the age group, *d* represents the causative disease, and *y* represents the year.

For ease of comprehension, we further defined the following parameters: blindness-related DALYs by age and causative disease (R); population size (A); population age structure (or population mean age) (B); the disease-specific prevalence rate of blindness for each age group (C); and the severity of blindness-causing diseases (D). Subsequently, we could simplify two time periods of interest (n = 1, 2) and estimate the changes in disease severity through the additive contribution of DALYs attributable to severity changes, as follows:

*R*_1_ = *A*_1_ × *B*_1_ × *C*_1_ × *D*_1_

*R*_2_ = *A*_2_ × *B*_2_ × *C*_2_ × *D*_2_

For these calculations, we assumed that *A*, *B*, and *C* remained the same over time by applying a standardised rate of *A*, *B*, and *C* [4].

Next, we calculated the additive contribution of disease severity changes to the change in blindness burden between periods (n = 1, 2) as follows:



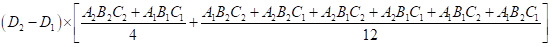


We then adopted the analytical method designed by Cheng et al. [13] to analyse the changes in severity of blindness-causing disease. The absolute contribution, i.e. attributable DALYs, refers to the number of DALYs attributed to disease severity changes. The relative contribution, i.e. attributable proportion, was calculated by dividing the number of DALYs attributable to changes in disease severity by the total number of DALYs in 1990.

Given our reliance on the comprehensive GBD 2019 data set, our study's capacity to discern significant changes in blindness-related DALYs attributable to severity changes is intrinsically linked to this dataset’s methodological strengths. Although traditional statistical power analysis cannot directly apply to our secondary data analysis approach, the extensive scope of GBD data underpins the robustness of our findings within the dataset’s well-established parameters.

We generated all calculations and graphs using R, version 4.2.2 (R Core Team, Vienna, Austria).

## RESULTS

### Global changes in blindness-related DALYs attributable to changes in disease severity

**Figure 1:**
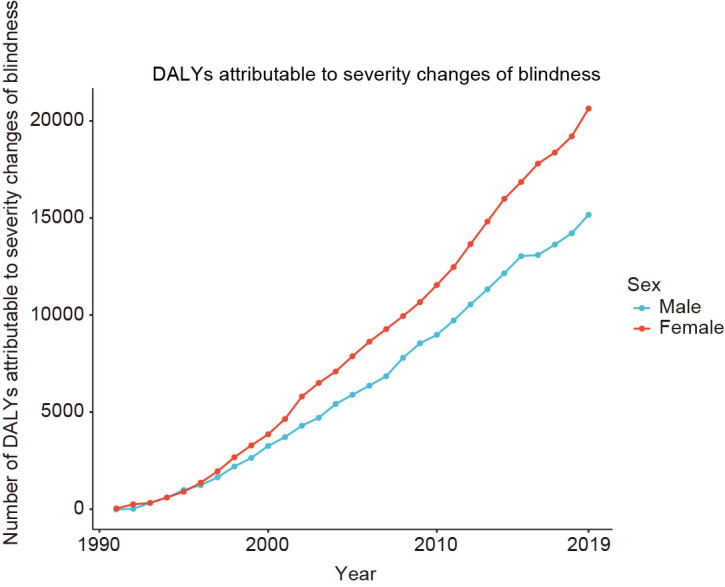
Global changes in blindness-related DALYs attributable to disease severity changes from 1991 to 2019. The decomposition was conducted using the number of blindness-related DALYs in 1990 as the reference for each year. DALYs – disability-adjusted life-years.

From 1991 to 2019, the number of blindness-related DALYs attributed to changes in disease severity increased considerably, reaching 15 165.11 in men and 20 639.32 in women (**Figure 1**). In general, the severity of most causes of blindness increased during the study period, but the impact of changes varied significantly across the 18 causes (**Table 1**). For example, the greatest increase in attributable DALYs was associated with changes in the severity of cataracts (n = 5054.29 for men, n = 8853.08 for women), refraction disorders (n = 1397.13 for men, n = 2098.99 for women), and glaucoma (n = 1289.89 for men, n = 1152.70 for women).

**Table 1:** Attributable DALYs and proportions associated with disease severity changes for specific blindness causes between 1990 and 2019

	Men		Women	
**Cause of blindness**	**DALYs attributable to severity changes**	**Attributable proportion (%)**	**DALYs attributable to severity changes**	**Attributable proportion (%)**
Cataract	5054.29	0.21	8853.08	0.32
Other vision loss	2440.17	0.10	3160.92	0.11
Refraction disorders	1397.13	0.06	2098.99	0.08
Glaucoma	1289.89	0.05	1152.70	0.04
Trachoma	821.48	0.03	490.40	0.02
Neonatal preterm birth	711.11	0.03	875.65	0.03
Age-related macular degeneration	472.08	0.02	473.05	0.02
Neonatal sepsis and other neonatal infections	211.32	0.01	126.63	<0.01
Diabetes mellitus type 2	111.95	<0.01	72.92	<0.01
Vitamin A deficiency	94.68	<0.01	191.27	0.01
Onchocerciasis	67.31	<0.01	49.70	<0.01
Encephalitis	22.44	<0.01	19.10	<0.01
Meningitis	20.45	<0.01	15.36	<0.01
Malaria	1.98	<0.01	1.74	<0.01
Haemolytic disease and other neonatal jaundice	0.27	<0.01	−0.29	<0
Tetanus	0.00	<0.01	0.00	<0.01
Diabetes mellitus type 1	−0.76	<0.01	3.15	<0.01
Neonatal encephalopathy due to birth asphyxia and trauma	−13.64	<0	8.37	<0.01

DALYs – disability-adjusted life-years

Notably, both attributable proportions and DALYs were greater in women than in men; although the ranking of the cause-specific decomposition for the former was marginally different from that for the later, their results were comparable.

### SDI-specific changes in blindness-related DALYs attributable to disease severity changes

We noted substantial increases in DALYs attributed to increased disease severity in all SDI groups between 1990 and 2019, with the largest increases observed in the low-, low-middle-, and middle-SDI groups, compared to relatively small increases in the high- and high-middle-SDI groups.

**Figure 2:**
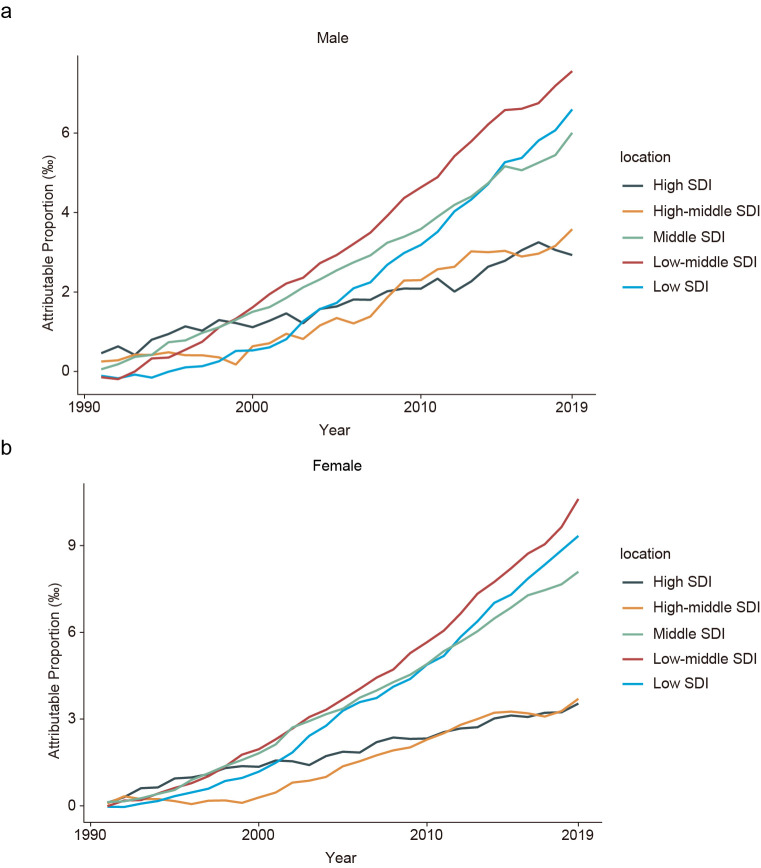
Changes in blindness-related DALYs attributable to disease severity changes across SDI regions. **Panel A.** Changes in men. **Panel B.** changes in women. The decomposition was conducted using the number of blindness-related DALYs in each region in 1990 as the reference for each year. DALYs – disability-adjusted life-years, SDI – sociodemographic index.

In general, these patterns were similar for both sexes, with attributable proportions in the high-, high-middle-, middle-, low-middle-, and low-SDI groups of 0.29% (n = 391.96), 0.36% (n = 1396.74), 0.60% (n = 5218.27), 0.76% (n = 5333.44), and 0.66% (n = 2121.63) among men, and 0.35% (n = 644.31), 0.37% (n = 1835.31), 0.81% (n = 8193.40), 1.06% (n = 8073.05), and 0.93% (n = 3125.78) among women, respectively (**Figure 2**).

In terms of the severity of specific diseases, we found a slight degree of heterogeneity across SDI groups, with substantial increases in severity observed for cataracts and glaucoma globally, and age-related macular degeneration (AMD) in the high-SDI group; for refraction disorders in the high-middle- and middle-SDI groups; and for trachoma in men and retinopathy of prematurity (ROP) in women in the low-middle- and low-SDI groups (Table S2 in the **Online Supplementary Document**).

### Region-specific changes in blindness-related DALYs attributable to disease severity changes

Between 1990 and 2019, changes in disease severity were associated with increases in blindness-related DALYs in all 21 geographic regions. Both Attributable DALY proportions for both men and women were the highest in South Asia (0.82%, DALY = 6381.37 and 1.15%, DALY = 9043.51, respectively) and the lowest in high-income North America (0.03%, DALY = 9.33 and 0.03%, DALY = 14.69, respectively) (**Table 2**).

**Table 2:** Attributable DALYs and proportions associated with disease severity changes for 21 geographic regions between 1990 and 2019

	Men		Women	
**Geographic region**	**DALYs attributable to severity changes**	**Attributable proportion (%)**	**DALYs attributable to severity changes**	**Attributable proportion (%)**
South Asia	6381.37	0.82	9043.51	1.15
Tropical Latin America	624.52	0.76	957.79	1.10
North Africa and Middle East	1086.50	0.61	1795.78	0.94
Andean Latin America	112.82	0.59	167.70	0.85
East Asia	2751.45	0.59	3922.08	0.77
Central sub-Saharan Africa	78.11	0.59	118.18	0.80
Eastern sub-Saharan Africa	561.18	0.57	926.98	0.83
Southeast Asia	1547.15	0.51	3231.47	0.74
Central Latin America	305.37	0.46	409.13	0.63
Australasia	14.53	0.45	20.82	0.50
Western sub-Saharan Africa	425.73	0.35	657.20	0.51
Caribbean	56.00	0.34	66.86	0.41
Oceania	5.68	0.30	5.96	0.28
High-income Asia Pacific	95.89	0.29	202.71	0.57
Southern Latin America	28.81	0.28	8.28	0.07
Central Asia	51.39	0.27	86.02	0.34
Southern Sub-Saharan Africa	66.04	0.24	123.65	0.38
Central Europe	50.94	0.23	63.36	0.22
Eastern Europe	77.83	0.15	206.54	0.21
Western Europe	128.10	0.15	376.33	0.26
High-income North America	9.33	0.03	14.69	0.03

DALYs – disability-adjusted life-years

### Country-specific changes in blindness-related DALYs attributable to disease severity changes

**Figure 3:**
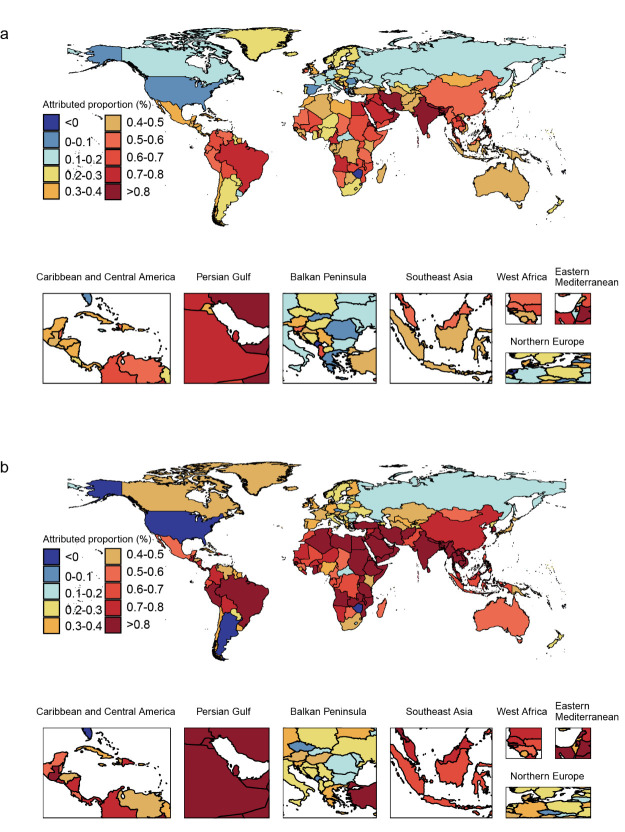
Changes in blindness-related DALYs attributable to disease severity changes in 204 countries and territories between 1990 and 2019. **Panel A.** Changes in men. **Panel B.** Changes in women. DALYs – disability-adjusted life-years.

From 1990 to 2019, disease severity changes were associated with increased blindness-related DALYs for both sexes in 199 of the 204 countries and territories. Attributable DALY proportions ranged from 1.30% in Equatorial Guinea to −0.07% in Zimbabwe for men and from 1.73% in Qatar to −0.06% in Argentina for women. Generally, higher attributable proportions were observed in less-developed countries and territories, and cross-country disparities were more pronounced for women than for men (**Figure 3**; Table S3 in the **Online Supplementary Document**).

## DISCUSSION

Increases in the severity of blindness-causing diseases between 1990 and 2019 were related to increases in attributable DALYs globally, with 15 165.11 DALYs in men and 20 639.32 DALYs in women in 2019. Disease severity increased primarily in low-, low-middle-, and middle-SDI countries. The largest increases in blindness-related DALYs attributable to changes in disease severity were observed for cataracts, refraction disorders, and glaucoma. Attributable DALYs and proportions were greater, and cross-country disparities were more evident for women than for men. Finally, the largest increases in blindness-related DALYs in high-SDI countries were attributed to cataracts, glaucoma, and AMD. The relative rankings of refraction disorders, trachoma, and ROP increased considerably with declining SDI.

Our study provides an exhaustive set of estimations regarding the evolving health effects of blindness. Previous research has reported that the age-standardised prevalence of blindness has decreased significantly worldwide [1]. What distinguishes our study from existing ones on the burden of blindness is that we evaluated global changes in blindness-related DALYs attributable to changes in disease severity, which are independent of prevalence, and that we examined disparities across sexes, causes, regions, and countries/territories to generate broader insights.

The variations in the severity of blindness-causing diseases can be explained by fluctuations in the distribution of the long-term consequences of these diseases over time. Human life expectancy has markedly increased at the global level, primarily due to advances in medical treatment. However, despite improved life expectancy, gaps remain in terms of healthy life expectancy. Globally, the average life expectancy increased significantly by six years, from 66.8 years in 2000 to 73.1 years in 2019 [14]. This rise in life expectancy can be attributed mainly to advances in maternal and child health, alongside substantial investments and enhancements in the management of communicable diseases, including HIV, tuberculosis, and malaria. Additionally, worldwide reductions in child stunting, alcohol consumption, and tobacco use, coupled with improved access to safely managed drinking water and sanitation, basic hygiene, and clean fuels and technologies for cooking, have played a crucial role. While the healthy life expectancy increased by 9%, from 58.1 years in 2000 to 63.5 in 2019, this was more a result of declining mortality rates than a decrease in years lived with disability [15,16]. Hence, this increase by 5.4 years did not match the increase in overall life expectancy (6.3 years). Health disparities, including indicators of eye health, likewise remain widespread [3].

In patients with blindness-causing diseases, the duration of the terminal disease phase is extended, resulting in increased severity over time. This increased severity suggests a shortfall in preventing blindness and providing social assistance after the onset of blindness. Moreover, our finding that the increase in disease severity is more pronounced in underdeveloped regions than in highly developed regions implies that not all countries have experienced the advantages of enhanced vision care. Additionally, the observed discrepancies between sexes suggest more unaddressed opportunities for improvement among women than among men.

We further observed increases in disease severity, predominantly for cataracts, refraction disorders, and glaucoma globally, as well as for AMD in high-SDI countries and for trachoma and ROP in lower-SDI countries specifically.

Cataracts and refraction disorders are major causes of avoidable blindness [17]. Interventions to correct uncorrected refractive errors and unoperated cataracts, meanwhile, are highly cost-effective [18]. Currently, the only feasible therapy for cataracts is surgery, which requires trained surgeons and health care systems equipped to perform operations and efficiently handle postoperative complications. Efforts to reduce the rates of unoperated cataracts have included large-scale campaigns, particularly in remote areas, and expanding surgical services in terms of capacity and accessibility. The sex discrepancy we observed in our study also presents a critical problem for minimising vision loss and for addressing sex injustices. Implementing outreach screening campaigns could increase the equity of access to health care services for vulnerable groups, such as women [19]. Hence, robust eye care infrastructures should be established, supplemented with community outreach activities as the primary interventions.

Refraction disorders may be efficiently treated with corrective methods, including eyeglasses, contact lenses, or refractive surgery. Since 1990, the frequency of uncorrected aphakia has significantly decreased owing to the widespread use of intraocular lenses after cataract surgery [20]. In contrast, the prevalence of myopia has increased significantly, particularly in urban areas [21]. The key measures for reducing the rates of uncorrected refractive errors include improving refractive services and providing low-cost eyeglasses. Quality-focussed interventions include strengthening government oversight and clinical regulation of the distribution of glasses, and adopting standardised refraction training programmes [22,23]. Quantity-focussed interventions, in turn, include increasing the number of persons skilled in dispensing glasses and performing refraction measurements, and the number of primary and community care access points. Furthermore, providing health care services with patient subsidies while accelerating the availability of cost-effective and high-quality goods is critical.

Glaucoma is the second leading cause of blindness and the leading cause of irreversible blindness. Glaucoma detection may be hindered in eyes with dense cataracts, possibly leading to underestimating the prevalence of glaucoma-related irreversible blindness. The efficacy of tonometry for glaucoma screening has not been established, and visual acuity measurement is not considered useful due to its limited ability to detect glaucomatous optic neuropathy in its early stages [24]. In most cases, once glaucoma is diagnosed, its development can be successfully slowed down using medications [25]. Therefore, it is critical to improve monitoring systems, raise awareness of the risk among affected persons’ families, and ensure the efficacy of therapy once treatment begins.

The severity of AMD has increased significantly in high-SDI countries, which may be attributed to a lag in effective treatment. Indeed, the age-standardised prevalence of AMD-related blindness has decreased by approximately 30%, likely due to the widespread implementation of anti-vascular endothelial growth factor treatment for exudative AMD [26]. However, many individuals have nonexudative AMD, which is currently hardly curable and can lead to irreversible blindness, necessitating targeted therapeutic interventions and preventive strategies for nonexudative AMD. Furthermore, epidemiological studies support the hypothesis that a healthy diet might prevent the onset of early AMD and hinder its progression [27]. This observation may help elucidate the factors contributing to increased AMD severity in high-SDI countries. Previous studies have reported that administering supplements, including vitamins C and E, zinc, lutein, and zeaxanthin, could slow the progression from moderate to advanced AMD in some individuals [28]. Specifically, nutritious dietary patterns such as the Mediterranean or low glycaemic index diets could protect against AMD [29,30], whereas Western dietary patterns or high glycaemic index diets increase the risk of developing AMD [30,31]. In addition to unhealthy diets, obesity poses a significant risk for the exacerbation of AMD in high-SDI nations; an increase of 0.1 in waist-to-hip ratio, a metric used to assess abdominal obesity, corresponded to a 13% increase in the possibility of early-onset AMD and a 75% increase in the possibility of late-onset AMD among men [32].

In lower-SDI countries, we observed increases in the severity of trachoma and ROP. Trachoma constituted a significant burden on the adult population. The transmission of *Chlamydia trachomatis* is associated with significant poverty, limited water use of less than 20 l per day, lack of soap availability or facial cleansing practices, lack of toilet utilisation, and a high density of flies [33]. Regarding ROP, the increase in newborn inpatient care, which varies in terms of quality, has brought less-developed countries to the verge of a severe ROP pandemic, with sub-Saharan Africa as an example [34]. Managing vision loss in newborns affected by ROP necessitates widespread availability of SpO2 monitoring and enhanced disease screening and treatment coverage.

This study has several limitations. First, the outcomes are contingent upon the accuracy and reliability of the data used. Although the GBD methodology and results are known to be cutting-edge, robust, and dependable, they are fundamentally limited by the quality of the available data. GBD data for specific areas are insufficiently robust and sparse, particularly for low- and low-middle-SDI areas, which may affect and underestimate disease burden. Ou reliance on aggregated data simplifies the complex interplay of factors influencing blindness, potentially overshadowing critical nuances such as regional disparities and individual variations in disease severity. Although this approach provides a broad overview, it is important to acknowledge that it may not fully reflect the diverse and localised impacts of changes in severity. Second, we could not obtain complete posterior samples of cause-specific blindness data stratified by age, sex, location, or year from the GBD 2019 study, which precluded us from generating 95% confidence intervals for our estimations. Third, changes in the severity of blindness-causing diseases are multifaceted phenomena. The methodology used in this study only considers four criteria, disregarding any heterogeneity underlying other related factors. Our analyses, specific to subpopulations and categorised by sex, SDI level, and cause of blindness, partly addressed this heterogeneity; however, we could not account for all heterogeneity. In addition, while our study offers significant insights into the dynamics of disease burden changes, the four-factor decomposition analysis method has limitations. Specifically, this study focussed on quantifying various factors’ relative contributions to changes in disease burden rather than inferring causality. Our findings contribute a novel perspective on understanding disease burden dynamics, but the validation of results presents challenges. Our findings should be interpreted and applied with an awareness of these limitations. The aforementioned constraints might be addressed by methodological advances and by improving the data quality in future studies.

Despite these limitations, our study provides valuable insights that may guide the development of vision health policies and the reformation of eye care systems. The increasing trend in the severity of blindness-causing diseases might signal a novel stage of social development and health transition. To successfully address these challenges, health resource allocation should target preventing blindness caused by diseases with the largest increases in severity and improving long-term social security and equality. Our results also help identify countries that have achieved excellent outcomes in managing blindness burden, which may be used for reference.

## CONCLUSIONS

We observed an increasing burden of blindness severity from 1990 to 2019, underscoring a global challenge in narrowing the gap between rising life expectancy and the effective management of diseases leading to blindness. Despite medical advances in the field of ophthalmology, our analysis indicates that strides in life expectancy have not been paralleled by progress in mitigating the severity of blindness, particularly in countries with lower SDIs and among women. This discrepancy highlights the urgent need for focussed preventive and therapeutic strategies to enhance visual health outcomes.

To effectively address this disparity, we propose several actionable recommendations for policymakers. First, comprehensive vision screening programmes should be implemented, especially in lower-SDI countries, to enhance the early identification and treatment of eye conditions. Second, investing in public health campaigns to raise awareness about preventable causes of blindness and promote eye health can profoundly impact women and vulnerable populations. Third, enhancing access to quality eye care services by increasing funding for ophthalmology services and subsidising treatment costs for low-income individuals could be an essential approach. Finally, encouraging research into cost-effective treatments for the most common causes of blindness will ensure that medical advancements translate into practical, accessible solutions for those most at risk. Policymakers can devise strategies that aim to extend life expectancy and significantly improve quality of life by improving visual health outcomes, aligning medical advancements with real-world needs.

## Additional material


Online Supplementary Document
