# Supplementary material for: Quantifying the impact of disease severity changes on the burden of blindness: A global decomposition analysis
Source: J Glob Health. 2024 Nov 1;14:04248. doi: 10.7189/jogh.14.04248 (PMC11529148; doi:10.7189/jogh.14.04248)
Supplement: Online Supplementary Document [file jogh-14-04248-s001.pdf]

Table S1. List of International Classification of Diseases (ICD) codes mapped to the causes of blindness.

Cause of blindness	ICD10 code	ICD10 code used in hospital/claims analyses	ICD9 code	ICD9 code used in hospital/claims analyses
Age-related macular degeneration	H35.3–H35.389	H35.3–H35.389	362.5–362.57	362.5–362.57
Cataract	H25–H26.9, H28–H28.8	H25–H28.8, Q12.0	366–366.9	366–366.9, 743.3–743.34
Diabetes mellitus type 1	E10–E10.11, E10.3–E10.9	E10–E10.11, E10.3–E10.9	250–250.0, 250.01, 250.03–250.1, 250.11, 250.13–250.2, 250.21, 250.23–250.3, 250.31, 250.33–250.39, 250.5, 250.51, 250.53–250.6, 250.61, 250.63–250.7, 250.71, 250.73–250.8, 250.81, 250.83–250.9, 250.91, 250.93–250.99	250–250.0, 250.01–250.01, 250.03–250.1, 250.11–250.11, 250.13–250.2, 250.21–250.21, 250.23–250.3, 250.31–250.31, 250.33–250.39, 250.5–250.5, 250.51–250.51, 250.53–250.6, 250.61–250.61, 250.63–250.7, 250.71–250.71, 250.73–250.8, 250.81–250.81, 250.83–250.9, 250.91–250.91, 250.93–250.99
Diabetes mellitus type 2	E11–E11.1, E11.3–E11.9	NA	250.00, 250.02, 250.10, 250.12, 250.20, 250.22, 250.30, 250.32, 250.50, 250.52,	NA

			250.60, 250.62, 250.70, 250.72, 250.80, 250.82, 250.90, 250.92	
Encephalitis	A83–A85.2, A85.8–A86.0, B94.1, F07.1, G04–G05.8, Z24.1	A83–A86.0, B94.1, F07.1, G04–G05.8	062–064.9, 310.89, 323–323.9, V05.0–V05.1	062–064.9, 310.89, 323–323.9
Glaucoma	H40–H40.9, H42–H42.8	H40–H42.8	365–365.9	365–365.9
Hemolytic disease and other neonatal jaundice	P55–P59.9	P55–P60.0	773–774.9	773–774.9, 776.2
Malaria	B50–B50.0, B50.8–B52.0, B52.8–B53.1, B53.8–B54.0, P37.3–P37.4	NA	084–084.9, V12.03, V75.1	NA
Meningitis	A39–A39.9, A87–A87.9, D86.81, G00–G03.9, G06–G09.9, Z20.811, Z22.31	A39–A39.9, A87–A87.9, G00–G03.9	036–036.9, 047–049.9, 054.72, 320–320.3, 320.5–322.9, 324–326.9, V01.84	036–036.9, 047–049.9, 054.72, 320–320.3, 320.5–322.9
Neonatal encephalopathy due to birth asphyxia and trauma	P02–P03.9, P10–P15.9, P20–P21.9, P24–P24.9, P52–P52.9, P90–P91.9	P21–P21.9, P24–P24.9, P91–P91.9	761.7–763.9, 767–768, 768.2–768.9, 779.0–779.2	NA
Neonatal preterm birth	P07.2–P07.39, P22–P22.9, P25–P28.9,	P05–P07.39	765.21–765.9, 769–770, 770.2–770.9,	764–765.9

	P61.2, P77–P77.9		776.6, 777.5–777.53	
Neonatal sepsis and other neonatal infections	A40.1, B95.1, P36–P36.9, P38–P39.9	P36–P36.9, P38–P39.9, P77–P78.1	041.02, 771, 771.4–771.89, V02.51	041.02, 771, 771.4–771.89, 777.5–777.7
Onchocerciasis	B73–B73.1	NA	125.3	NA
Other vision loss	H27–H27.9, H31–H35.23, H35.4–H36.8, H46–H51.9, H53–H54.9	H31–H35.23, H35.4–H36.8, H46–H54.9	360.8–362, 362.1–362.43, 362.6–363.9, 368–369.9, 377–378.9	361–362, 362.1–362.43, 362.6–363.9, 367–369.9, 377–378.9
Refraction disorders	H52–H52.7	NA	367–367.9	NA
Tetanus	A33–A35.0, Z23.5	A33–A35.0	037–037.9, 771.3, V03.7	037–037.9, 771.3
Trachoma	A71–A71.9, A74.0, B94.0	A71–A74.0, B94.0	076–076.9, V73.6	076–076.9, V73.6
Vitamin A deficiency	E50–E50.9, E64.1	E50.0–E50.7	264–264.9	264.0–264.6

Table S2. Changes in blindness-related DALYs associated with severity changes by specific cause and SDI region between 1990 and 2019.

SDI Region	Men			Women		
	Cause of blindness	DALYs attributable to severity changes	Attributed Proportion (‰)	Cause of blindness	DALYs attributable to severity changes	Attributed Proportion (‰)
High SDI	Cataract	113.41	0.85	Cataract	22.00	1.13
	Other vision loss	91.20	0.68	Glaucoma	148.52	0.81

	Glaucoma	83.85	0.63	Age-related macular degeneration	99.15	0.54
	Age-related macular degeneration	14.98	0.11	Other vision loss	73.75	0.40
	Refraction disorders	11.61	0.09	Refraction disorders	37.30	0.20
	Diabetes mellitus type 2	8.85	0.07	Neonatal preterm birth	8.37	0.05
	Neonatal encephalopathy due to birth asphyxia and trauma	2.67	0.02	Neonatal encephalopathy due to birth asphyxia and trauma	5.42	0.03
	Neonatal sepsis and other neonatal infections	2.04	0.02	Diabetes mellitus type 2	1.75	0.01
	Diabetes mellitus type 1	0.08	0.00	Diabetes mellitus type 1	0.07	0.00
	Malaria	0.01	0.00	Vitamin A deficiency	0.03	0.00
	Trachoma	0.00	0.00	Malaria	0.01	0.00
	Tetanus	0.00	0.00	Meningitis	0.00	0.00
	Encephalitis	-0.01	0.00	Encephalitis	0.00	0.00
	Meningitis	-0.02	0.00	Trachoma	0.00	0.00
	Vitamin A deficiency	-0.03	0.00	Tetanus	0.00	0.00
	Hemolytic disease and other neonatal jaundice	-0.07	0.00	Hemolytic disease and other neonatal jaundice	-0.06	0.00
	Neonatal preterm birth	-2.60	-0.02	Neonatal sepsis and other neonatal infections	-0.07	0.00
High middle SDI	Cataract	406.26	1.04	Cataract	738.45	1.49

	Other vision loss	332.26	0.85	Other vision loss	436.35	0.88
	Glaucoma	191.96	0.49	Glaucoma	186.27	0.38
	Refraction disorders	106.48	0.27	Refraction disorders	124.23	0.25
	Neonatal preterm birth	87.57	0.22	Age-related macular degeneration	65.28	0.13
	Age-related macular degeneration	56.44	0.14	Neonatal preterm birth	39.36	0.08
	Neonatal sepsis and other neonatal infections	38.99	0.10	Diabetes mellitus type 2	10.53	0.02
	Diabetes mellitus type 2	30.65	0.08	Neonatal sepsis and other neonatal infections	9.60	0.02
	Trachoma	13.32	0.03	Trachoma	7.06	0.01
	Encephalitis	0.30	0.00	Neonatal encephalopathy due to birth asphyxia and trauma	4.97	0.01
	Malaria	0.02	0.00	Diabetes mellitus type 1	2.08	0.00
	Meningitis	0.02	0.00	Vitamin A deficiency	1.62	0.00
	Tetanus	0.00	0.00	Onchocerciasis	1.28	0.00
	Hemolytic disease and other neonatal jaundice	-0.43	0.00	Encephalitis	0.17	0.00
	Neonatal encephalopathy due to birth asphyxia and trauma	-0.45	0.00	Malaria	0.02	0.00
	Onchocerciasis	-0.80	0.00	Meningitis	0.01	0.00

	Vitamin A deficiency	-0.89	0.00	Tetanus	0.00	0.00
	Diabetes mellitus type 1	-1.51	0.00	Hemolytic disease and other neonatal jaundice	-0.63	0.00
Middle SDI	Cataract	1689.27	1.94	Cataract	3294.75	3.25
	Other vision loss	1269.43	1.46	Other vision loss	1885.17	1.86
	Refraction disorders	524.65	0.60	Refraction disorders	693.38	0.69
	Glaucoma	507.36	0.58	Glaucoma	560.15	0.55
	Neonatal preterm birth	245.32	0.28	Age-related macular degeneration	323.71	0.32
	Age-related macular degeneration	234.05	0.27	Neonatal preterm birth	307.91	0.30
	Neonatal sepsis and other neonatal infections	121.46	0.14	Diabetes mellitus type 2	119.60	0.12
	Trachoma	115.32	0.13	Neonatal sepsis and other neonatal infections	83.66	0.08
	Diabetes mellitus type 2	62.53	0.07	Vitamin A deficiency	75.23	0.07
	Vitamin A deficiency	22.32	0.03	Trachoma	47.02	0.05
	Onchocerciasis	7.68	0.01	Encephalitis	4.66	0.00
	Encephalitis	5.69	0.01	Onchocerciasis	2.42	0.00
	Meningitis	2.39	0.00	Meningitis	1.54	0.00
	Malaria	0.25	0.00	Malaria	0.26	0.00
	Diabetes mellitus type 1	0.15	0.00	Diabetes mellitus type 1	0.24	0.00
	Hemolytic disease and other neonatal jaundice	0.04	0.00	Hemolytic disease and other neonatal jaundice	0.19	0.00

	Tetanus	0.00	0.00	Tetanus	0.00	0.00
	Neonatal encephalopathy due to birth asphyxia and trauma	-3.20	0.00	Neonatal encephalopathy due to birth asphyxia and trauma	-0.25	0.00
Low middle SDI	Cataract	2120.47	3.01	Cataract	3711.89	4.88
	Other vision loss	739.74	1.05	Other vision loss	1045.73	1.37
	Refraction disorders	493.06	0.70	Refraction disorders	887.30	1.17
	Trachoma	414.90	0.59	Neonatal preterm birth	439.56	0.58
	Glaucoma	398.55	0.56	Glaucoma	394.71	0.52
	Neonatal preterm birth	310.69	0.44	Age-related macular degeneration	272.98	0.36
	Age-related macular degeneration	169.58	0.24	Trachoma	258.94	0.34
	Vitamin A deficiency	65.19	0.09	Vitamin A deficiency	115.41	0.15
	Neonatal sepsis and other neonatal infections	53.85	0.08	Diabetes mellitus type 2	54.18	0.07
	Diabetes mellitus type 2	23.47	0.03	Neonatal sepsis and other neonatal infections	36.55	0.05
	Encephalitis	14.20	0.02	Onchocerciasis	20.92	0.03
	Onchocerciasis	6.76	0.01	Encephalitis	12.10	0.02
	Meningitis	6.66	0.01	Meningitis	5.20	0.01
	Hemolytic disease and other neonatal jaundice	0.78	0.00	Malaria	0.65	0.00

	Malaria	0.67	0.00	Hemolytic disease and other neonatal jaundice	0.56	0.00
	Diabetes mellitus type 1	0.33	0.00	Neonatal encephalopathy due to birth asphyxia and trauma	0.12	0.00
	Tetanus	0.00	0.00	Diabetes mellitus type 1	0.03	0.00
	Neonatal encephalopathy due to birth asphyxia and trauma	-1.66	0.00	Tetanus	0.00	0.00
Low SDI	Cataract	650.05	2.02	Cataract	1203.67	3.59
	Trachoma	313.32	0.97	Other vision loss	461.18	1.38
	Other vision loss	289.98	0.90	Neonatal preterm birth	268.61	0.80
	Refraction disorders	162.88	0.51	Trachoma	245.56	0.73
	Glaucoma	150.86	0.47	Refraction disorders	233.90	0.70
	Neonatal preterm birth	138.15	0.43	Glaucoma	199.72	0.60
	Onchocerciasis	57.94	0.18	Age-related macular degeneration	73.83	0.22
	Vitamin A deficiency	42.40	0.13	Vitamin A deficiency	47.77	0.14
	Age-related macular degeneration	35.32	0.11	Neonatal sepsis and other neonatal infections	33.21	0.10
	Neonatal sepsis and other neonatal infections	29.92	0.09	Onchocerciasis	32.69	0.10
	Meningitis	15.66	0.05	Meningitis	13.19	0.04

	Neonatal encephalopathy due to birth asphyxia and trauma	5.21	0.02	Neonatal encephalopathy due to birth asphyxia and trauma	6.76	0.02
	Diabetes mellitus type 2	5.05	0.02	Encephalitis	3.51	0.01
	Encephalitis	2.41	0.01	Malaria	0.99	0.00
	Malaria	1.20	0.00	Hemolytic disease and other neonatal jaundice	0.33	0.00
	Hemolytic disease and other neonatal jaundice	0.43	0.00	Tetanus	0.00	0.00
	Diabetes mellitus type 1	0.04	0.00	Diabetes mellitus type 1	-0.01	0.00
	Tetanus	0.00	0.00	Diabetes mellitus type 2	-0.53	0.00

DALYs, disability-adjusted life-years, SDI, sociodemographic index.

Table S3. Changes in blindness-related DALYs associated with severity changes in 204 countries and territories between 1990 and 2019.

**Men**			**Women**		
**Country**	**DALYs attributable to severity changes**	**Attributed Proportion (‰)**	**Country**	**DALYs attributable to severity changes**	**Attributed Proportion (‰)**
Equatorial Guinea	7.18	12.99	Qatar	1.61	17.29

United Arab Emirates	6.59	11.11	Maldives	0.76	14.31
Nepal	85.63	10.47	Jordan	12.36	13.84
Maldives	0.54	9.81	Bahrain	1.98	13.32
Bangladesh	465.84	9.62	Equatorial Guinea	8.75	12.43
Eritrea	12.52	8.92	Bangladesh	788.60	12.30
Bhutan	1.36	8.84	India	7642.65	12.13
Timor-Leste	4.23	8.81	Iran (Islamic Republic of)	369.27	11.50
Jordan	6.70	8.71	Guatemala	54.02	11.36
India	5513.01	8.66	Brazil	953.99	11.21
Djibouti	1.28	8.42	Somalia	38.51	11.14
Oman	11.94	8.30	Viet Nam	572.14	11.13
Iran (Islamic Republic of)	250.41	8.02	Angola	45.52	10.92
Angola	31.62	7.80	Bhutan	1.91	10.92
Brazil	621.52	7.70	Nepal	98.32	10.76
Bahrain	1.37	7.62	Zambia	21.60	10.72
Lao People's Democratic Republic	5.05	7.53	Coted'Ivoire	52.34	10.56
Democratic People's Republic of Korea	8.25	7.39	United Arab Emirates	3.26	10.47
Qatar	1.29	7.37	Eritrea	18.22	10.28
Singapore	3.51	7.32	Afghanistan	138.80	10.25
Egypt	191.47	7.23	Lebanon	21.23	10.14
Cameroon	32.78	7.15	Algeria	130.50	10.04
Iraq	58.18	7.10	Iraq	92.64	9.95

Comoros	1.50	7.09	United Republic of Tanzania	160.88	9.93
Saudi Arabia	114.39	7.08	Kuwait	3.49	9.91
Syrian Arab Republic	40.53	6.86	Sudan	142.15	9.75
Togo	9.54	6.86	Mozambique	77.50	9.45
Mozambique	42.30	6.72	Cambodia	96.11	9.40
Bolivia (Plurinational State of)	20.88	6.67	Timor-Leste	4.80	9.36
Burundi	6.55	6.51	Turkey	238.28	9.36
Ethiopia	232.17	6.49	Ethiopia	385.54	9.35
Uganda	27.41	6.37	Myanmar	390.03	9.22
Burkina Faso	31.91	6.32	Uganda	47.29	9.15
Malawi	26.78	6.30	Palestine	9.66	9.13
Colombia	87.55	6.30	Peru	123.94	9.05
Mali	57.03	6.23	Libya	18.18	9.03
Zambia	19.01	6.23	Oman	5.53	9.00
Yemen	33.57	6.19	Malawi	41.92	8.81
Albania	2.97	6.17	Ghana	56.08	8.76
Sudan	93.22	6.09	Thailand	218.91	8.71
Thailand	128.27	6.01	Saudi Arabia	139.48	8.62
Slovenia	1.77	5.98	Yemen	56.29	8.52
Peru	73.69	5.96	Egypt	255.59	8.48
Rwanda	9.87	5.96	Republic of Korea	90.76	8.30
China	2742.92	5.94	Lao People's Democratic Republic	11.04	8.16
Malaysia	54.03	5.89	Rwanda	13.20	8.09

United Republic of Tanzania	85.79	5.84	Sri Lanka	52.23	8.08
Cambodia	38.72	5.80	Tunisia	44.17	7.95
Ecuador	19.92	5.70	Malaysia	77.88	7.93
Taiwan (Province of China)	2.01	5.66	Djibouti	1.13	7.89
Belize	0.32	5.63	China	3925.77	7.78
Venezuela (Bolivarian Republic of)	43.14	5.63	Dominican Republic	19.28	7.78
Somalia	16.99	5.42	Burkina Faso	46.73	7.76
Sri Lanka	24.74	5.32	El Salvador	21.59	7.71
Senegal	20.36	5.30	Mauritania	9.80	7.55
Palestine	3.16	5.25	Guinea-Bissau	4.68	7.52
Cyprus	0.62	5.20	Bolivia (Plurinational State of)	23.93	7.47
Myanmar	151.74	5.19	Taiwan (Province of China)	4.19	7.46
Mauritania	5.48	5.14	Brunei Darussalam	0.28	7.45
Saint Lucia	0.21	5.12	Syrian Arab Republic	42.88	7.37
Ireland	2.57	5.08	Liberia	10.11	7.35
Malta	0.28	5.08	Nicaragua	11.98	7.34
Haiti	13.65	5.06	Comoros	1.72	7.33
Namibia	4.33	5.03	Colombia	101.28	7.28
Chad	19.52	5.03	Niger	32.93	7.27
Liberia	7.10	5.02	Senegal	36.54	7.23
Philippines	102.76	5.00	Namibia	6.48	7.19

Viet Nam	142.97	4.99	Panama	8.42	7.09
Croatia	3.89	4.99	Benin	20.93	6.93
Guatemala	19.58	4.96	Democratic Republic of the Congo	49.53	6.78
Australia	13.00	4.92	Gabon	3.05	6.76
Indonesia	879.16	4.89	Philippines	172.75	6.61
Madagascar	16.06	4.87	Pakistan	526.42	6.54
Gabon	1.72	4.86	Morocco	65.59	6.54
Algeria	57.16	4.85	Ecuador	18.05	6.47
Congo	3.16	4.84	Congo	5.08	6.46
Tunisia	25.67	4.81	Mali	59.70	6.22
Democratic Republic of the Congo	31.30	4.81	Indonesia	1621.49	6.20
Benin	12.05	4.72	Gambia	2.95	6.16
Turkey	109.54	4.72	Togo	10.30	6.15
Pakistan	384.56	4.70	Sao Tome and Principe	0.46	5.94
Kenya	59.94	4.65	Guinea	28.39	5.90
Afghanistan	49.47	4.62	Mongolia	5.44	5.82
Republic of Korea	35.12	4.61	Belize	0.33	5.79
Turkmenistan	2.92	4.53	Australia	19.17	5.76
Coted'Ivoire	23.11	4.53	Saint Lucia	0.28	5.69
Papua New Guinea	5.68	4.49	Cameroon	32.51	5.69
South Sudan	26.41	4.49	Costa Rica	7.08	5.64
Tajikistan	7.24	4.47	Mexico	164.23	5.59
Mauritius	1.65	4.47	Madagascar	19.98	5.58

San Marino	0.02	4.42	Mauritius	3.25	5.47
Botswana	3.92	4.39	Chad	24.11	5.35
Bermuda	0.08	4.39	Botswana	4.36	5.26
Sierra Leone	9.63	4.33	Kenya	75.64	4.99
Sao Tome and Principe	0.28	4.30	Seychelles	0.28	4.96
Guinea	18.65	4.28	Portugal	14.96	4.95
Lebanon	9.02	4.26	Japan	117.21	4.90
Andorra	0.03	4.15	Honduras	9.09	4.85
Bahamas	0.25	4.14	Singapore	2.83	4.74
El Salvador	15.37	4.11	United States Virgin Islands	0.17	4.72
Armenia	4.38	4.08	Venezuela (Bolivarian Republic of)	40.31	4.68
Guinea-Bissau	2.03	3.99	Haiti	13.03	4.63
Libya	9.59	3.93	Turkmenistan	3.58	4.53
Kyrgyzstan	4.90	3.93	Kazakhstan	29.82	4.51
Kuwait	1.91	3.91	Canada	15.76	4.48
Nicaragua	5.59	3.85	Northern Mariana Islands	0.03	4.48
Panama	5.66	3.84	South Africa	109.04	4.36
Dominican Republic	11.48	3.82	Bahamas	0.32	4.34
Monaco	0.02	3.80	Austria	9.62	4.32
Serbia	7.34	3.74	Iceland	0.44	4.30
Suriname	0.68	3.73	Belgium	12.08	4.29
Chile	10.66	3.69	South Sudan	28.27	4.27
United Kingdom	33.08	3.65	Cyprus	0.65	4.26

Austria	4.30	3.64	France	49.89	4.22
Puerto Rico	4.23	3.55	Trinidad and Tobago	1.66	4.21
Mexico	108.81	3.53	Cabo Verde	1.03	4.21
Azerbaijan	7.30	3.45	Greenland	0.03	4.18
Gambia	1.48	3.43	Saint Vincent and the Grenadines	0.17	4.15
Cabo Verde	0.57	3.42	Denmark	4.11	4.14
Cuba	24.52	3.37	Hungary	16.74	4.06
Lithuania	2.56	3.36	Papua New Guinea	5.75	4.06
Dominica	0.09	3.15	Ireland	2.93	3.98
Mongolia	2.82	3.14	Cuba	27.51	3.97
Denmark	2.46	3.09	Israel	3.71	3.96
Saint Vincent and the Grenadines	0.10	3.06	Uzbekistan	26.53	3.94
Honduras	4.90	3.06	Suriname	0.83	3.86
United States Virgin Islands	0.10	3.05	Chile	11.47	3.86
Estonia	0.45	3.03	Slovenia	1.67	3.83
Morocco	26.81	3.01	Andorra	0.03	3.76
Hungary	8.16	2.95	Eswatini	1.51	3.73
Trinidad and Tobago	1.04	2.94	United Kingdom	54.52	3.68
Ghana	16.14	2.93	Greece	8.66	3.65
Costa Rica	3.27	2.91	Saint Kitts and Nevis	0.06	3.62
Grenada	0.10	2.90	Burundi	4.15	3.46
South Africa	55.57	2.74	Puerto Rico	4.64	3.44
Guyana	0.63	2.73	Sierra Leone	6.67	3.40

New Zealand	1.67	2.71	Guyana	0.80	3.32
Czechia	4.69	2.67	Armenia	4.25	3.27
Argentina	18.30	2.62	Spain	68.96	3.21
Vanuatu	0.06	2.61	Solomon Islands	0.31	3.18
Niger	11.06	2.54	Finland	4.55	3.16
Japan	63.37	2.51	Belarus	13.07	3.14
Northern Mariana Islands	0.02	2.51	Jamaica	2.82	3.12
Lesotho	2.90	2.49	Malta	0.25	3.06
Bosnia and Herzegovina	1.92	2.40	Uruguay	1.94	3.05
Nigeria	160.32	2.39	Germany	71.54	3.04
Marshall Islands	0.03	2.37	Nigeria	203.16	3.02
North Macedonia	0.86	2.36	Kyrgyzstan	5.74	3.02
Portugal	4.53	2.35	Azerbaijan	8.52	2.98
Norway	1.45	2.33	Netherlands	9.31	2.93
Switzerland	2.31	2.33	Tuvalu	0.01	2.93
Samoa	0.09	2.33	Marshall Islands	0.04	2.88
Belgium	3.85	2.33	Cook Islands	0.03	2.83
Poland	13.57	2.32	Latvia	3.10	2.83
Finland	2.22	2.28	Norway	2.49	2.82
Paraguay	4.19	2.21	Ukraine	61.56	2.82
Niue	0.00	2.15	Switzerland	4.69	2.80
Greenland	0.01	2.14	Paraguay	6.07	2.76
Georgia	3.87	2.12	Grenada	0.13	2.71
Uzbekistan	11.36	2.08	Albania	1.42	2.68
Iceland	0.11	2.05	Estonia	1.16	2.64

Sweden	3.09	2.00	Democratic People's Republic of Korea	6.87	2.63
Uruguay	0.91	1.98	North Macedonia	1.00	2.58
Cook Islands	0.02	1.96	Tajikistan	4.76	2.52
Israel	1.35	1.92	Croatia	2.79	2.52
France	8.81	1.84	Bermuda	0.06	2.48
Kazakhstan	8.13	1.84	Niue	0.00	2.42
Luxembourg	0.10	1.74	Samoa	0.12	2.40
Nauru	0.00	1.73	New Zealand	1.84	2.31
Tuvalu	0.01	1.71	Poland	17.19	2.14
Saint Kitts and Nevis	0.02	1.70	Sweden	5.48	2.07
Russian Federation	55.41	1.63	Serbia	4.75	2.07
Ukraine	17.86	1.59	Italy	98.25	2.07
Belarus	3.31	1.50	Bosnia and Herzegovina	2.00	2.06
Kiribati	0.03	1.35	Barbados	0.11	2.05
Jamaica	1.02	1.30	Romania	11.08	1.89
Bulgaria	2.12	1.29	Dominica	0.07	1.89
Republic of Moldova	2.11	1.28	Luxembourg	0.18	1.88
Barbados	0.04	1.26	Monaco	0.02	1.88
Tokelau	0.00	1.24	Russian Federation	126.82	1.87
Central African Republic	1.29	1.16	Bulgaria	3.49	1.87
Seychelles	0.03	1.14	Lesotho	2.52	1.80
Antigua and Barbuda	0.02	1.13	Tokelau	0.00	1.77
Italy	33.67	1.07	Micronesia (Federated States of)	0.06	1.71

Micronesia (Federated States of)	0.03	1.05	Central African Republic	2.18	1.67
Canada	2.96	1.04	Guam	0.05	1.67
Germany	12.61	1.01	Republic of Moldova	2.20	1.32
Tonga	0.02	0.89	Slovakia	1.46	1.24
Palau	0.00	0.85	Tonga	0.03	1.20
Romania	3.70	0.77	Nauru	0.00	1.20
Eswatini	0.26	0.76	Lithuania	1.54	1.18
Latvia	0.40	0.72	Palau	0.00	1.15
Spain	5.68	0.48	American Samoa	0.01	1.13
Greece	0.66	0.37	Montenegro	0.10	0.86
Brunei Darussalam	0.01	0.33	Antigua and Barbuda	0.02	0.71
Guam	0.01	0.26	Kiribati	0.01	0.49
Slovakia	0.10	0.11	Georgia	1.23	0.46
United States of America	3.25	0.11	Czechia	1.08	0.42
Solomon Islands	0.00	0.05	Fiji	0.11	0.39
Netherlands	-0.04	-0.02	United States of America	-5.13	-0.13
Fiji	-0.09	-0.38	San Marino	0.00	-0.17
American Samoa	0.00	-0.45	Zimbabwe	-1.78	-0.44
Montenegro	-0.05	-0.50	Vanuatu	-0.01	-0.45
Zimbabwe	-2.63	-0.74	Argentina	-4.95	-0.62

DALYs, disability-adjusted life-years.
